# Molecular Characteristics, Virulence Gene and Wall Teichoic Acid Glycosyltransferase Profiles of *Staphylococcus aureus*: A Multicenter Study in China

**DOI:** 10.3389/fmicb.2020.02013

**Published:** 2020-08-19

**Authors:** Mengyuan Xiong, Jin Zhao, Tao Huang, Weihua Wang, Lijun Wang, Zhijun Zhao, Xuehan Li, Junying Zhou, Xiao Xiao, Yunbao Pan, Jun Lin, Yirong Li

**Affiliations:** ^1^Department of Laboratory Medicine, Zhongnan Hospital of Wuhan University, Wuhan, China; ^2^Department of Laboratory Medicine, Hainan General Hospital, Haikou, China; ^3^Department of Laboratory Medicine, The Affiliated Hospital of Medical School of Ningbo University, Ningbo, China; ^4^Department of Laboratory Medicine, Beijing Tsinghua Changgung Hospital, Beijing, China; ^5^Department of Laboratory Medicine, General Hospital of Ningxia Medical University, Yinchuan, China; ^6^Department of Gastroenterology, Zhongnan Hospital of Wuhan University, Wuhan, China

**Keywords:** *Staphylococcus aureus*, molecular characterization, virulence gene, wall choice acid, glycosyltransferase

## Abstract

*Staphylococcus aureus* (*S. aureus*) constantly evolves under host and environment pressures. The monitoring network is essential in assessing the epidemiology of *S. aureus* infections. A total of 555 *S. aureus* isolates were collected from five hospitals in three different geographical regions of China for the investigation of molecular characteristics, antibiotic resistance, virulence gene, and wall teichoic acid (WTA) glycosyltransferase gene profiles. 233 (42.0%) isolates were identified as MRSA, and 323 (58.2%) were defined as multidrug-resistant (MDR) isolates. MRSA prevalence showed no significant difference among the three regions. In contrast, the MDR prevalence was significantly higher in central China than that in northern China (63.5% vs. 50.8%, *P* < 0.05). Thirty-eight sequence types (STs) belonging to 17 clone complexes (CCs) and 126 distinct *spa*-types were identified. The most prevalent clone was ST59-t437 (9.7%, 54/555), followed by ST22-t309 (7.6%, 42/555) and ST5-t2460 (7.2%, 40/555). Most ST59-t437 and ST5-t2460 were MRSA isolates, whereas most ST22-t309 was MSSA isolates. The predominant clones varied in different geographical areas. The distribution of the *pvl*, *etb*, *tsst*, *clfb*, *sdrC*, *sdrD*, *hlg*, *fnbA*, and *hla* genes showed significant differences among different regions. We found five WTA glycosyltransferase gene profiles, with *tarP*-/*tarS*+/*tarM*-/*tagN*- being the most common combination. Remarkably, the *tarP* gene was identified in more CCs than just CC5 and CC398. All of 16 *tarP*-positive isolates also contained the *tarS*. Moreover, *tarS* was present in almost all *S. aureus* isolates except 10 ST630 isolates. The *tagN* gene was only detected in 10 of 12 ST630 *S. aureus* isolates without *tarS*. The *tarM* gene was absent in CC5 and CC398. In brief, there were regional differences among molecular characteristics, antibiotic resistance, and virulence gene profiles. The *tarS*-negative ST630 lineage carried the *tagN*, which was never found before, indicating that it may be capable of expressing GroP-α-GalNAc WTA and exchanging mobile genetic elements with coagulase-negative staphylococci (CoNS).

## Introduction

*Staphylococcus aureus* (*S. aureus*), a Gram-positive coccus, generally colonizes on the surface of skin and mucous membranes and is thought to be one of the most significant human opportunistic pathogens in both community and hospital settings. *S. aureus* can cause diverse diseases, ranging from superficial lesions to life-threatening endocarditis, bone and joint infections, sepsis, pneumonia, and toxic shock syndrome (Liu et al., 2016). The mortality rate of *S. aureus* invasive infection was extremely high (>80%) before the emergence of effective antibiotics (Peacock and Paterson, 2015). The introduction of β-lactam antibiotics has significantly reduced the mortality rate and improved prognosis since the early 1940s. However, *S. aureus* is capable of evolving and adapting under the selective pressure of antibiotics, resulting in the rapid emergence of methicillin-resistant *S. aureus* (MRSA) and multidrug-resistant strains (Manara et al., 2018). Vancomycin and linezolid generally serve as agents for therapy of invasive MRSA infections due to the side effects. Cefazolin and a series of antistaphylococcal penicillins including methicillin, nafcillin, oxacillin, cloxacillin, and dicloxacillin are considered as the treatment for methicillin-susceptible *S. aureus* (MSSA) infection. Because the results of antimicrobial susceptibility test (AST) are not available within 24 to 72 h, initial therapy for *S. aureus* infection is often empiric and guided by the urgency of the situation. Empiric antimicrobial therapy requires regular investigation of antimicrobial resistance profiles focusing on a country or region, especially the prevalence of MRSA. It was reported that some certain molecular types of *S. aureus* were significantly associated with methicillin-resistant status: most ST188-t189 isolates were found to be MSSA, whereas the majority of ST59-t437 and ST5-t2460 isolates were found to be MRSA. Some certain molecular types were also reported to be associated with the occurrence of complications, severity, and mortality of infection. One study showed that ST398 MSSA was associated with high mortality (Bouiller et al., 2016). ST121, a globally disseminated highly virulent clone, often caused longer hospitalization and prolonged antimicrobial therapy (Rao et al., 2015). Moreover, another study found that clone complex 30 (CC30) and CC5 were easier to develop hematogenous complications (Chen et al., 2010). The capacity of *S. aureus* to successfully cause damage to hosts is mainly dependent upon the carriage of a number of virulence factors, which contribute to adhesion, colonization, destruction of local tissue, and evasion of host immune responses (Gordon and Lowy, 2008; Otto, 2010). Therefore, it is of great significance to monitor molecular characteristics, virulence genes, and drug resistance of *S. aureus* in specific areas which may help to predict the change of prevalence of MRSA and prognosis of *S. aureus* infection.

Mobile genetic elements (MGEs) encoding resistance and virulence genes have been proved to be widespread in *S. aureus* which can be mobilized and exchanged by horizontal gene transfer (HGT) to shape bacterial genome plasticity and allow rapid adaptation to the changing environmental challenges (Thomas and Nielsen, 2005). Given the rarity of genetic conjugation loci and restriction against transformation, generalized transduction via bacteriophages is considered the main pattern of horizontal gene transfer of MGEs in *S. aureus* (Lindsay, 2010; Xia and Wolz, 2014). The glycosylated WTA is the receptor of bacteriophage and contributes to the intraspecies and interspecies transfers of MGEs, including staphylococcal cassette chromosome *mec* (SCC*mec*) (Winstel et al., 2013). It is reasonable to speculate that the antimicrobial resistance and virulence gene profiles are related to the glycosylated patterns of WTA. WTA structures are highly variable among Gram-positive bacteria and are usually species or strain-specific (Neuhaus and Baddiley, 2003). Most *S. aureus* strains express poly ribitol-phosphate (poly RboP) WTA with up to 40 repeating units, modified with D-alanine (D-Ala) and α-, β-N-acetylglucosamine (GlcNAc) at the O-2 position and O-4 position of ribitol residue, respectively (Brown et al., 2013). Recently, *S. aureus* ST395 lineage has been proved to produce a unique coagulase-negative staphylococci (CoNS)-like WTA, which is composed of glycerol-phosphate (GroP) backbone modified with α-N-acetyl-D-galactosamine (α-GalNAc) (Winstel et al., 2014). To date, four glycosyltransferases including TarM, TarS, TarP, and TagN were found to be involved in the glycosylation of WTA individually or collectively. TarM was responsible for α-GlcNAc glycosylation at the O-4 position of the ribitol residue, whereas TarS and TarP were required for the β-GlcNAc glycosylation at the O-4 and O-3 position, respectively (Xia et al., 2010; Brown et al., 2012; Gerlach et al., 2018). TagN was identified as an α-GalNAc glycosyltransferase in the special ST395 lineage (Winstel et al., 2014). High-throughput WTA structure analysis requires purified WTA specimens and nuclear magnetic resonance (NMR) spectroscopy, making the identification of WTA glycosylation patterns both time-consuming and laborious. The presence of the glycosyltransferase genes in *S. aureus* isolates which can be easily detected by PCR has been generally used to infer their WTA glycosylation patterns (Mistretta et al., 2019). Therefore, the investigation of WTA glycosyltransferase gene profiles may contribute to understanding the genetic diversification and the emergence of new pathogenic strains in *S. aureus*.

## Materials and Methods

### *S. aureus* Isolates

A total of 555 non-duplicate *S. aureus* isolates were collected from five hospitals in three geographical regions (northern, central, southern China) from January 2017 to June 2018 (Table 1). These isolates were collected from inpatients with infectious symptoms, and only the first identified isolate from each patient was collected. All isolates were initially identified by colony morphology, Gram staining, coagulase tests at the clinical microbiology laboratory, and then confirmed by matrix-assisted laser desorption/ionization-time of flight (MALDI-TOF) mass spectrometry (VITEK^®^ MS, bioMérieux). These strains were isolated from various specimens including sputum and pharynx swabs (*n* = 194, 35.0%), wound secretion (*n* = 134, 24.1%), pus (*n* = 123, 22.2%), blood (*n* = 44, 7.9%), and others (bronchoalveolar lavage fluid, bile, catheter tip, stool, marrow, cerebrospinal fluid, ascites, joint fluid, urine, penile secretion, drainage fluid, tissue) (*n* = 60, 10.8%). In general, the isolation of *S. aureus* from normally sterile body sites was defined as invasive infection. Normally sterile body sites included the blood, pericardial fluid, cerebrospinal fluid, joint/synovial fluid, pleural fluid, bone, or other normally sterile sites. All identified isolates were stored in Tryptic Soy Broth (TSB) with 15% glycerol at −80°C for further analysis.

**TABLE 1:** The distribution of *S. aureus* isolates collected in this study.

Region	Hospital	No. of *S. aureus* isolates	No. of MRSA isolates (%)
Northern China	NX	84	26 (31.0)
	BJ	42	18 (42.9)
Southern China	HN	54	20 (37.3)
	NB	90	44 (48.9)
Central China	WH	285	125 (43.9)
Total		555	233 (42.0)

*NX, General Hospital Of Ningxia Medical University; BJ, Beijing Tsinghua Changgung Hospital; HN, Hainan General Hospital; NB, The Affiliated Hospital Of Medical School Of Ningbo University; WH, Zhongnan Hospital Of Wuhan University.*

### *S. aureus* DNA Extraction

Each *S. aureus* isolate was cultured on a blood agar plate at 37°C overnight and then a single colony was transferred into 2 mL TSB medium to enrich for 16 h at 220 rpm. DNA of *S. aureus* was extracted using a rapid DNA extraction kit (Aidlab, China) according to the manufacturer’s instructions with 1 mg/ml lysostaphin (Sigma, China) and DNA concentration was determined using the NanoDrop 2000 (Thermo Fisher Scientific, United States). The extracted DNAs were all stored at −20°C and prepared for the next experiments.

### MLST and *spa* Typing

All isolates were characterized by the combination of *Staphylococcal protein A* (*spa*) typing and multilocus sequence typing (MLST) as previously described (Li et al., 2018). Briefly, the *spa* polymorphic X repeat region of *S. aureus* isolates was amplified by PCR and sequenced with sanger dideoxy DNA sequencing (Tianyi Huiyuan, China). The *spa* types were assigned through the Ridom web server (http://spaserver.ridom.de) (Koreen et al., 2004). MLST is a sequence-based genotyping method based on seven known *S. aureus* housekeeping genes (*arcC*, *glpF*, *pta*, *aroE*, *yqiL*, *gmk*, and *tpi*). The sequence type and clone complex were determined through the MLST online database (https://pubmlst.org/saureus/) and eBURST, respectively. A Minimum Spanning Tree (MST) based on ST types was constructed using BioNumerics 7 software (Applied Maths, Belgium).

### PCR Assays for Genotyping

Four WTA glycosyltransferase genes (*tarS, tarM, tarP*, and *tagN*) and sixteen virulence genes were examined using PCR assays, including the Panton-Valentine leukocidin (*pvl*), the hemolysin (*hlg, hla*, and *hlb*), the adhesion genes (*fnbA*, *fnbB*, *clfA*, *clfB, sdrC*, *sdrD*, *sdrE*, *icaA*, and *cna*), the exfoliative toxin (*eta* and *etb*), and the toxic shock syndrome toxin-1 (*tsst*). To verify the designation of the USA300 genotype, all the isolates were also screened for the presence of arginine catabolic mobile element (ACME). ACME-arcA and ACME-opp3AB genes were used as markers of the ACME-arc cluster and the ACME-opp3 cluster, respectively (Diep et al., 2006b, 2008). The PCR reaction pools consisted of 10 μl 2xTaq Master Mix (Tianyi Huiyuan, China), 1 μl forward primer (10 μM), 1 μl reverse primer (10 μM), 1 μl DNA template (200 ng/μl–600 ng/μl), and 7 μl ddH_2_O. The PCR conditions were determined and adjusted according to primers and amplicons length. All the primers used in this study were chemically synthesized by Tianyi Huiyuan (Wuhan, China) (Supplementary Table S1). All PCR products were analyzed by 1% agarose gel electrophoresis.

### Antimicrobial Susceptibility Testing

Antimicrobial susceptibility tests were performed using the automated VITEK 2^®^ Compact system (Biomérieux, France). Fourteen antibiotics were involved in this study, including levofloxacin (LEV), moxifloxacin (MXF), ciprofloxacin (CIP), gentamicin (GEN), penicillin (PEN), vancomycin (VAN), erythromycin (ERY), clindamycin (CLI), tetracycline (TET), rifampicin (RIF), oxacillin (OXA), quinupristin-dalfopristin (QUD), tigecycline (TGC), and linezolid (LZD). The MRSA isolate was further identified using PCR to confirm the presence of the *mecA*. ATCC 25923 and 29213 were employed to be the quality control strains for AST. The Clinical and Laboratory Standards Institute (CLSI) M100-S27 was used to explain AST results. Isolates that were resistant to three or more kinds of antibiotics were defined as multidrug-resistant (MDR).

### Statistical Analysis

IBM SPSS Statistics 25.0 was used for all data analyses. Chi-squared or Fisher exact tests were used to analyze virulence genes and antimicrobial resistance differences in *S. aureus* isolates with regard to the geographical regions. The significance level was set at 0.05. Bonferroni corrections (corrected α^∗^ = 0.05/3 = 0.017) were applied to correct the *P*-values of multiple comparisons to control inflation of the type I error rate.

## Results

### Molecular Characteristics of *S. aureus* Isolates

**FIGURE 1:**
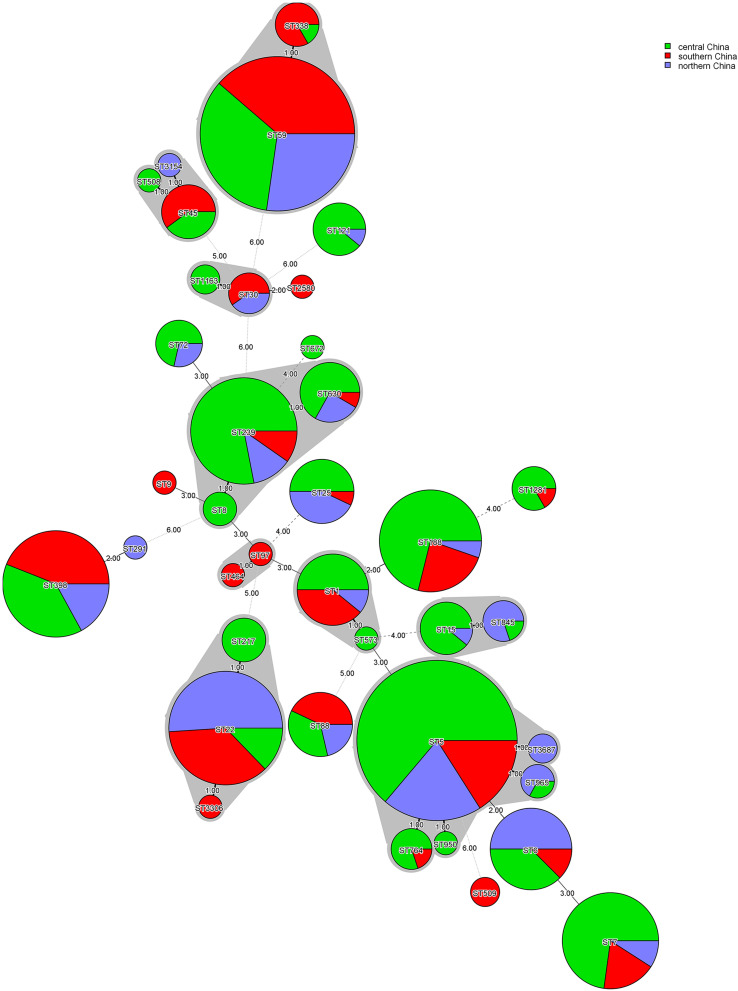
Minimum-spanning tree, as constructed from the MLST data of 555 *S. aureus* isolates collected from three different regions in China. Circle sizes represent the number of isolates, and circle areas are colored by geographical region source and labeled with sequence types (STs). The different colors represented isolates from Central China (*n* = 285), Southern China (*n* = 144), and Northern China (*n* = 126).

The MLST typing of the 555 *S. aureus* isolates revealed a total of 38 distinct ST types. ST5 was most prevalent (16.9%, 94/555), followed by ST59 (15.9%, 88/555), ST22 (8.5%, 47/555), ST239 (7.4%, 41/555), ST398 (7.4%, 41/555), ST188 (6.8%, 38/555), ST7 (5.9%, 33/555), and other STs accounted for 31.2%. Seventeen CC types were identified according to eBURST, namely CC5, CC59, CC8, CC1, CC22, CC398, CC7, CC15, CC25, CC88, CC45, CC121, CC30, CC20, CC97, CC509, and CC291. A total of 54 isolates were defined as invasive isolates, of which 24 were MRSA isolates (Supplementary Tables S2, S3). The regions of different ST isolates were shown in Figure 1. The most prevalent CCs among all strains were CC5, CC59, CC8, CC1, and CC22, representing 71.9% of all clones. Specifically, the three most abundant CCs in the MRSA group were CC59 (32.6%), CC5 (27.9%), and CC8 (19.7%), while the most common clone in the MSSA group was CC5 (20.2%), followed by CC1 (16.1%), and CC22 (14.3%). The major epidemic ST in MRSA isolates was ST59 (30.5%), followed by ST5 (25.3%), ST239 (16.3%), and ST45 (4.3%). In contrast, ST22 (12.7%) and ST188 (11.5%) were the most prevalent STs in. MSSA isolates, followed by ST5 (10.9%), ST398 (10.2%), and ST7 (8.7%). By spa typing, 126 distinct types were found, with t437 (10.1%), t309 (8.5%), t2460 (7.2%), t030 (5.9%) and t189 (5.9%) being the most predominant. When spa typing and MSLT were combined to analyze, the most prevalent clones were ST59-t437 (9.7%, 54/555), followed by ST22-t309 (7.6%, 42/555), ST5-t2460 (7.2%, 40/555), ST239-t030 (5.9%, 33/555), and ST188-t189 (5.9%, 33/555). It was found that there was a strong association between some certain spa types and STs: ST22 was associated mainly with t309 (89.4%, 42/47), ST188 was primarily associated with t189 (86.8%, 33/38), and ST239 was mainly associated with t030 (80.5%, 33/41). In addition, most ST59-t437 and ST5-t2460 were MRSA isolates, whereas most ST22-t309 were MSSA isolates, suggesting that certain molecular types of *S. aureus* were also significantly associated with methicillin-resistant status. Remarkably, there were regional differences in *S. aureus* epidemic genotypes. ST5-t2460 and ST239-t030 were most common in central China, whereas ST22-t309 and ST59-t437 were most predominant in northern China. Besides, ST59, ST398, and ST22 were the main STs in southern China, with no significant difference in *spa* types.

### Antimicrobial Susceptibility

Detailed results of AST were listed in Table 2. All *S. aureus* isolates were sensitive to VAN, LZD, and TGC, but most isolates were resistant to PEN (91.7%, 509/555). The resistant rates to the remaining antibiotics ranged from 0.2% to 62.0%. There were 26 (4.7%) isolates that were susceptible to all tested antibiotics, and 103 (18.6%) isolates that were only resistant to PEN. Of 555 *S. aureus* isolates, 233 (42.0%) were assigned to be MRSA according to OXA susceptibility and FOX screening test. Compared to the MSSA isolates, MRSA isolates had higher resistant rates to the remaining ten antibiotics except for VAN, LZD, QUD, and TGC. The prevalence of MRSA showed no significant difference among three geographical regions (*P* > 0.05). There were regional differences in resistance rates to LEV, CIP, MXF, GEN, PEN, CLI, TET, and RIF. A total of 323 (58.2%) isolates were defined as MDR. The MDR prevalence of the MRSA isolates was significantly higher than that of the MSSA isolates (79.8% vs. 42.5%, *P* < 0.05). The prevalence of MDR in central China was significantly higher than that in northern China (63.5% vs. 50.8%, *P* < 0.017). Besides, there was no significant difference in MDR prevalence between central and southern China (63.5% vs. 54.2%, *P* > 0.017), southern and northern China (54.2 % vs. 50.8 %, *P* > 0.017).

**TABLE 2:** Antimicrobial resistance profiles of MRSA, MSSA, MDR, and *S. aureus* isolates in different regions.

Antibiotics	*S. aureus*	MRSA	MSSA	*P*-value^a^	Invasive	Non-invasive	*P*-value^b^	Northern China	Southern China	Central China	*P*-value^c^
											
	*n* = 555 (%)	*n* = 233 (%)	*n* = 322 (%)		*n* = 54 (%)	*n* = 501 (%)		*n* = 126 (%)	*n* = 144 (%)	*n* = 285 (%)	
Levofloxacin	23.2	45.9	6.8	0.000	24.1	23.2	0.879	19.8	11.8	30.5	0.000
Ciprofloxacin	24.5	48.5	7.1	0.000	24.1	24.6	0.938	20.6	16.0	30.5	0.002
Moxifloxacin	21.3	42.5	5.9	0.000	22.2	21.2	0.856	17.5	9.0	29.1	0.000
Gentamicin	16.6	29.6	7.1	0.000	20.4	16.2	0.430	11.1	8.3	23.2	0.000
Penicillin	91.7	100.0	85.7	0.000	92.6	91.6	1.000	86.5	92.4	93.7	0.049
Oxacillin	42.0	100.0	0.0	0.000	44.4	41.7	0.700	34.9	44.4	43.9	0.187
Vancomycin	0.0	0.0	0.0	–	0.0	0.0	–	0.0	0.0	0.0	–
Erythromycin	62.0	78.1	50.3	0.000	51.9	63.1	0.107	61.9	61.1	62.5	0.964
Clindamycin	54.6	68.2	44.7	0.000	51.9	54.9	0.670	42.1	55.6	59.6	0.004
Linezolid	0.0	0.0	0.0	–	0.0	0.0	–	0.0	0.0	0.0	–
Tetracycline	26.1	47.2	10.9	0.000	33.3	25.3	0.204	23.0	16.7	32.3	0.002
Rifampicin	8.6	19.7	0.6	0.000	7.4	8.8	0.931	4.8	4.2	12.6	0.003
Quinupristin-dalfopristin	0.2	0.4	0.0	0.871	0.0	0.2	1.000	0.8	0.0	0.0	0.227
Tigecycline	0.0	0.0	0.0	–	0.0	0.0	–	0.0	0.0	0.0	–
MDR	58.2	79.8	42.5	0.000	51.9	58.9	0.320	50.8	54.2	63.5	0.029

*^a^The antimicrobial resistances among MRSA were compared with those among MSSA isolates. ^b^The antimicrobial resistances among invasive isolates were compared with those among non-invasive isolates. ^c^Comparison of antimicrobial resistance across all three regions simultaneously.*

### Screening of Virulence Genes, ACME-arcA, and ACME-opp3AB Genes

**FIGURE 2:**
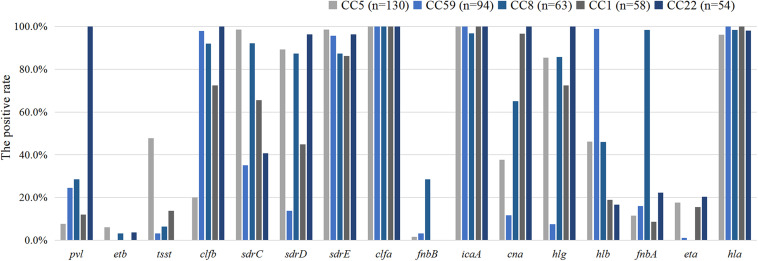
The distribution of virulence genes among the major clone complexes.

The pathogenicity of *S. aureus* largely depends on the carriage of plenty of virulence factors (Diep et al., 2006a). The frequencies of virulence genes detected in 555 *S. aureus* isolates were shown in Table 3. Virulence genes detected in at least 50% of all tested isolates included *clfa* (100%), *icaA* (99.6%), *hla* (91.5%), *sdrE* (85.6%), *clfb* (75.9%), *hlg* (74.2%), *sdrC* (65.6%), *sdrD* (62.7%), and *cna* (55.1 %). Multiple isolates also harbored *hlb* (41.3%), *pvl* (25.9%), *fnbA* (23.4%), *fnbB* (20.7%), *tst* (17.5%), *eta* (12.3%), and *etb* (5.6%). The frequencies of *fnbA*, *tsst*, *sdrE*, and *hlb* were significantly higher in the MRSA isolates than those in the MSSA isolates, whereas *pvl*, *etb*, *clfB*, *sdrD*, *fnbB*, *cna*, *hlg*, and *eta* were significantly lower. Four hundred and eleven (74.1%) *S. aureus* isolates carried eight or more virulence genes, of which 11 contained 12 virulence genes, 44 harbored 11, 109 contained 10, 128 carried 9, and 119 carried 8. The comparison of virulence genes among the major clone complexes (CC5, CC59, CC8, CC1, and CC22) was shown in Figure 2. In the present study, a higher carriage of fibronectin-binding protein genes (*fnbA* and *fnbB*) were found among CC8 isolates, while a higher carriage of *tsst* was found among CC5 isolates. All CC1 and CC22 isolates were not carrying *fnbB*, while all CC22 isolates carried *pvl*. The proportion of *hlb* among CC59 isolates was high, while the positive rates of *hlg*, *tsst*, and *cna* were 7.4%, 3.2%, and 11.7%, respectively. The serine-aspartate repeat protein-encoding *sdr* (CDE) genes were also correlated with the different MRSA lineages. 95.0% (527/555) of *S. aureus* isolates carried at least one *sdr* locus and 74.4% (413/555) possessed more than two *sdr* loci. Compared with the higher prevalence (>85.0%) of *sdrC* and *sdrD* among CC5 and CC8 isolates, the carriage rates of these two *sdr* loci among CC59 isolates were significantly lower, especially *sdrD* prevalence of 13.8%. Surprisingly, 86.9% of CC5 isolates were concomitantly positive for *sdrC*, *sdrD* and *sdrE*, indicating a significant correlation between concomitant carriage of the three *sdr* genes and CC5 isolates. The distribution of virulence genes also showed significant differences in different regions, including *pvl*, *etb*, *tsst*, *clfb*, *sdrC*, *sdrD*, *hlg*, *fnbA*, and *hla*. Remarkably, ACME-arcA and ACME-opp3AB were not found in the present study.

**TABLE 3:** The frequencies of virulence genes among *S. aureus*, MRSA and MSSA isolates.

Virulence genes	*S. aureus*	MRSA	MSSA	*P*-value^a^	invasive	Non-invasive	*P*-value^b^	Northern China	Southern China	Central China	*P*-value^c^
											
	*n* = 555 (%)	*n* = 233 (%)	*n* = 322 (%)		*n* = 54 (%)	*n* = 501 (%)		*n* = 126 (%)	*n* = 144 (%)	*n* = 285 (%)	
*pvl*	25.9	21.0	29.5	0.025	22.2	26.3	0.511	31.7	34.7	18.9	0.000
*etb*	5.6	2.6	7.8	0.009	3.7	5.8	0.747	13.5	6.9	1.4	0.000
*tsst*	17.5	30.0	8.4	0.000	18.5	17.4	0.832	15.9	9.7	22.1	0.005
*clfb*	75.9	70.4	79.8	0.010	70.4	76.4	0.322	82.5	85.4	68.1	0.000
*sdrC*	65.6	65.2	65.8	0.883	68.5	65.3	0.633	68.3	47.9	73.3	0.000
*sdrD*	62.7	56.7	67.1	0.012	63.0	62.7	0.967	64.3	48.6	69.1	0.000
*sdrE*	85.6	89.7	82.6	0.019	87.0	85.4	0.749	92.1	82.6	84.2	0.057
*clfa*	100.0	100.0	100.0	–	100.0	100.0	–	100.0	100.0	100.0	–
*fnbB*	20.7	11.2	27.6	0.000	14.8	21.4	0.260	20.6	22.9	19.6	0.733
*icaA*	99.6	100.0	99.4	0.626	100.0	99.6	1.000	100.0	100.0	99.3	0.733
*cna*	55.1	39.9	66.1	0.000	44.4	56.3	0.096	53.2	59.7	53.7	0.435
*hlg*	74.2	59.2	85.1	0.000	77.8	73.9	0.531	81.0	66.0	75.4	0.016
*hlb*	41.3	65.7	23.6	0.000	46.3	40.7	0.429	38.9	40.3	42.8	0.729
*fnbA*	23.4	35.2	14.9	0.000	22.2	23.6	0.826	11.9	34.0	23.2	0.000
*eta*	12.3	6.0	16.8	0.000	13.0	12.2	0.867	11.9	16.0	10.5	0.265
*hla*	91.5	92.3	91.0	0.593	88.9	91.8	0.633	97.6	89.6	89.8	0.020

*^a^The positive rates of virulence genes among MRSA were compared with those among MSSA isolates. ^b^The positive rates of virulence genes among invasive isolates were compared with those among non-invasive isolates. ^c^Comparison of virulence genes across all three regions simultaneously.*

### Detection of WTA Glycosyltransferase Genes

We found five patterns of WTA glycosylation in this study: *tarP*+/*tarS*+/*tarM*+/*tagN*-; *tarP*+/*tarS*+/*tarM*-/*tagN*-; *tarP*-/ *tarS*+/*tarM*+/*tagN*-; *tarP*-/*tarS*+/*tarM*-/*tagN*-; *tarP*-/*tarS*-/ *tarM*+/*tagN*+, accounting for 0.3%, 2.5%, 17.7%, 77.7% and 1.8%, respectively (Supplementary Table S4). One hundred and ten isolates (19.8%) belonged to various clone complexes harbored *tarM* genes. Sixteen isolates were found *tarP* positive, which existed not only in CC5 and CC398 but also in other clone complexes including CC97, CC1, CC15, and CC7 (Table 4). All *tarP*-positive isolates also contained *tarS.* The *tarS* gene was found in almost all 555 *S. aureus* strains, except in one special lineage ST630. We collected twelve ST630 isolates from four different hospitals, two contained *tarS*, while the other ten did not (Table 5). Remarkably, the unique WTA glycosyltransferase gene *tagN* was only detected in the ten *tarS*-negative ST630 *S. aureus* strains, indicating that the *tarS*-negative ST630 clones may have the similar GroP-GalNAc WTA structures as the ST395 clones. Moreover, all 12 ST630 isolates were *tarM* positive, which indicated that the ST630 lineage also presented the classical RboP-GlcNAc WTA.

**TABLE 4:** 16 *tarP* positive *S. aureus* strains.

Strain number.	Source	Molecular characteristics			WTA glycosyltransferase genes			
		SPA	ST	Clone Complex	*tarP*	*tarS*	*tarM*	*tagN*
HN303	Pus	t3904	ST464	CC97	+	+	–	–
HN311	Pus	t034	ST398	CC398	+	+	–	–
HN326	Pus	t189	ST188	CC1	+	+	–	–
HN328	Wound secretion	t034	ST398	CC398	+	+	–	–
NX21	Pus	t084	ST845	CC15	+	+	+	–
NX45	Pus	t084	ST845	CC15	+	+	+	–
NB87	Sputum	t091	ST7	CC7	+	+	–	–
NB89	Sputum	t091	ST7	CC7	+	+	–	–
WH6	Sputum	t091	ST7	CC7	+	+	–	–
WH48	Wound secretion	t189	ST188	CC1	+	+	–	–
WH49	Wound secretion	t189	ST188	CC1	+	+	–	–
WH53	Bronchoalveolar lavage fluid	t091	ST7	CC7	+	+	–	–
WH149	Sputum	t091	ST7	CC7	+	+	–	–
WH173	Sputum	t189	ST188	CC1	+	+	–	–
WH231	Wound secretion	t002	ST5	CC5	+	+	–	–
WH318	Urine	t002	ST5	CC5	+	+	–	–

**TABLE 5:** 12 ST630 *S. aureus* isolates collected in this study.

Strain number	Source	Molecular characteristics			WTA glycosyltransferase genes			
		SPA	ST	Clone Complex	*tarP*	*tarS*	*tarM*	*tagN*
BJ12	Pharynx swab	t377	ST630	CC8	–	+	+	–
BJ95	Blood	t377	ST630	CC8	–	–	+	+
HN288	Pus	t377	ST630	CC8	–	–	+	+
NX98	Tissue	t4549	ST630	CC8	–	–	+	+
WH39	Wound secretion	t2196	ST630	CC8	–	–	+	+
WH52	Wound secretion	t377	ST630	CC8	–	+	+	–
WH60	Wound secretion	t4549	ST630	CC8	–	–	+	+
WH99	Wound secretion	t4549	ST630	CC8	–	–	+	+
WH114	Catheter tip	t4047	ST630	CC8	–	–	+	+
WH119	Blood	t4549	ST630	CC8	–	–	+	+
WH211	Wound secretion	t4549	ST630	CC8	–	–	+	+
WH299	Wound secretion	t377	ST630	CC8	–	–	+	+

## Discussion

*Staphylococcus aureus* is one of the most frequently antimicrobial-resistant pathogens worldwide and some distinct lineages have been ecologically highly successful. A total of 555 *S. aureus* isolates were collected from five hospitals distributed in three geographical regions of China for epidemiological investigation in this study. Based on the *spa*-typing and MLST results, 38 STs and 126 spa types were found, indicating there were high diversity and complexity in their genetic background. The most prevalent reported *S. aureus* lineages are diverse and include ST239 (Goudarzi et al., 2017), ST5 (Li et al., 2018), ST59 (Ding et al., 2018), and ST188 (He et al., 2013) according to different resources, regions, ages, and diseases. In our study, the most predominant STs were ST5, ST59, ST22, ST239, and ST398, comprising over 50% of *S. aureus* isolates. Moreover, a strong association was found between some certain spa types and STs. In the present study, both ST5-t2460 and ST239-t030 were the most common clones in MRSA isolates, which was primarily attributable to their high prevalence in the central region. ST188-t189, like ST22-t309, was identified to be one of the most predominant clones in MSSA isolates, which was consistent with previous studies in central and southern China (Li et al., 2018; Yuan et al., 2019). We then analyzed the composition ratio and found only ST59-t437 occupied the top five positions in three regions indicating ST59-t437 is now a widely distributed *S. aureus* clone in China. ST22 was the predominant clone of both MSSA, and community-acquired MRSA (CA-MRSA) isolates collecting from Beijing and Urumqi (Xiao et al., 2019; Yuan et al., 2019), indicating that ST22-t309-MSSA was the emerging and most common clone in southern China as well as northern China. Moreover, the majority of the MSSA clones observed in the present study are globally disseminated. The frequencies of ST6, ST7, and ST398 were relatively high in the current study, indicating that some pandemic clones have arisen across the country. We found that ST1, ST5, ST8, ST630, ST398, and ST22 included both MSSA and MRSA, suggesting that these MRSA isolates probably evolved from extant MSSA isolates through the transfer of SCCmec element. The local variation in *S. aureus* lineages epidemic patterns emphasizes the importance of establishing a national multicenter antimicrobial monitoring network. As we all know, various virulence factors are closely related to the pathogenesis of *S. aureus.* For instance, *pvl* is a key virulence factor of *S. aureus*, mainly associated with mucosal necrotic lesions and soft tissue infections (SSTIs). In this study, *pvl* was detected in 144 *S. aureus* isolates (49 MRSA and 95 MSSA) and was most frequently in isolates collected from pus (*n* = 56, 38.9%), sputum (*n* = 38, 26.4%), and wound secretion (*n* = 33, 22.9%). Particularly, we found all CC22 isolates were *pvl* positive. The *sdr* proteins are members of a family of surface adhesions and have different roles in the pathogenicity of *S. aureus*. It has been shown that *sdrC* contributes to both bacterial adherence and biofilm formation (Barbu et al., 2014). *SdrD* played an important role in the host and pathogen immune response (Sitkiewicz et al., 2011). It is of interest to note that the frequencies of *tsst, sdr loci* in CC5 isolates were significantly higher than in other clones. The distribution of virulence genes indicated that different *S. aureus* lineages had specific virulence genes patterns. There was no significant difference in the prevalence of MRSA among the three regions. The significant difference in the resistance rates to some other antibiotics among different regions indicates that different geographical regions had their unique antimicrobial resistance profiles. This may be associated with the selection of antibiotics in the process of infection control across these regions. Therefore it is required to summarize and analyze the results of AST periodically for providing objective evidence of empirical antibiotic treatment. Previous findings have confirmed that there is an unexpected inverse correlation between disease severity and bacterial toxicity, and the expression of the *mecA* gene reduces bacterial virulence (Lindsay, 2010; He et al., 2013; Laabei et al., 2015). These results suggested that we should further consider how *S. aureus* balanced the relationship between virulence and drug resistance. USA300 was first identified based on its pulsed-field gel electrophoresis (PFGE) pattern in the United States, and soon became the dominant CA-MRSA circulating in North America (McDougal et al., 2003; Tenover and Goering, 2009; Nimmo, 2012). USA300 commonly are described as ST8, SCCmec IV, *pvl*-positive, and possess the ACME (Diep et al., 2006b, 2008; Murai et al., 2019). ST8 is very rare in Asia (Chuang and Huang, 2013), with sporadic findings of USA300 reported from South Korea (Jung et al., 2016) and Japan (Murai et al., 2019). Three ST8 isolates belonged to *spa*-type t7087 and t024 were found in the present study and the ACME was not identified. Although USA300 was not found in the current study, continued surveillance was still required due to its high virulence and transmissibility.

WTA accounts for about 60% of the *S. aureus* cell’s total dry weight and plays an important role in a variety of physiological processes. There are usually four types of glycosylated WTA in *S. aureus*: RboP-β-1,4-GlcNAc WTA (TarS-WTA), RboP-α-1,4-GlcNAc WTA (TarM-WTA), RboP-β-1,3-GlcNAc WTA (TarP-WTA) and GroP-α-GalNAc (TagN-WTA). Based on the different *S. aureus* strains, the configuration of the glycosidic linkage to the WTA repeat unit can be exclusively α-GlcNAc, exclusively β-GlcNAc, or a mixture of these two anomers. This is an extensive investigation of various WTA glycosylation patterns from different *S. aureus* strains by detecting the representative glycosyltransferase genes. The predominate glycosylation pattern in the present study is *tarP*-/*tarS*+/*tarM*-/*tagN*-, accounting for 77.7%. Of note, glycosylated WTA acts as bacteriophage receptors to mediate interspecies and intergeneric HGT and takes a great part in the pathogen-host immune response. Therefore, WTA has become an attractive drug target to the infection control of *S. aureus* and other Gram-positive pathogens in recent decades (Winstel et al., 2015; Mistretta et al., 2019; van Dalen et al., 2019). Gerlach et al. demonstrated that *tarP* played a crucial role in the fitness of these lineages and raised concerns of further dissemination by horizontal gene transfer, but the role of this novel RboP-β-1,3-GlcNAc WTA epitope in immune response remained controversial. Gerlach et al. concluded that this novel WTA epitope was essential for the ability of *S. aureus* to evade host immune defenses due to its poor immunogenicity and antibody recognition (Gerlach et al., 2018). On the contrary, Dalen et al. considered TarP-WTA as a highly dominant *S. aureus* antigen (van Dalen et al., 2019). We found that *tarP* also existed in other clones besides CC5 and CC398, suggesting that the *tarP* gene might have a broader coverage among *S. aureus*. Of note, we found 10 of 12 ST630 isolates which carried *tagN* instead of *tarS*. The *tarS*-positive ST630 isolates expressed a classical RboP-WTA with α/β-1,4-GlcNAc modification, whereas the *tarS-*negative ST630 isolates expressed both RboP-α-1,4-GlcNAc WTA and GroP-α-GalNAc WTA. TagN-WTA was not found exclusively in the ST395 lineage, and it may be more widespread in *S. aureus*. No significant difference was found in the distribution of virulence genes and antibiotics resistances among ST630 isolates collected from different sources. Remarkably, it has been revealed that β-GlcNAc WTA modification is required for PBP2a-mediated β-lactam resistance and the so-called SCCmec elements, or parts of them can be exchanged by HGT via bacteriophages between different *S. aureus* strains (Brown et al., 2012). The ST395 lineage expressing GroP-α-GalNAc WTA was unable to undergo HGT with typical *S. aureus* but may have an increased capacity for acquiring mobile genetic elements, including SCCmec and some virulence genes, from CoNS. ST630 clone has been reported in many previous studies (Yu et al., 2015; Liu et al., 2017), but none of them have described their special WTA phenotypes. We hypothesize that the special *tarP*-/*tarS*-/*tarM*+/*tagN*+ ST630 clone might serve as a hub for the continuous HGT of multiple MGEs such as antimicrobial resistance and pathogenicity islands (SaPIs) between both *S. aureus* and CoNS. In addition, it remained unclear why there were two types of ST630 and whether double WTA structure affected bacterial adhesion, pathogenicity, and phage susceptibility, which still needed further investigation. Furthermore, it has been reported that the transcription level of WTA glycosyltransferase genes can be different and strongly influenced by different environments in both *S. aureus* and other Gram-positive bacteria (Minnig et al., 2005; Mistretta et al., 2019). The Newman strain produces almost exclusively α-GlcNAc WTA (>90%) under *in vitro* normal growth conditions despite the presence of both the *tarM* and *tarS* genes (Mistretta et al., 2019). The WTA β-glycosylation has been reported at either the O-3 position or the O-4 position 4 of ribitol, which was mediated by *tarP* and *tarS*, respectively. Moreover, NMR analysis revealed that GlcNAc was almost exclusively attached to RboP C3 in *S. aureus* N315 which bears both *tarP* and *tarS*, suggesting that *tarP* is dominant over *tarS* (Gerlach et al., 2018). These findings indicate that individual genetic characteristics may not exactly reflect the WTA phenotypes and emphasize the importance of determining the actual WTA glycosylation patterns. This study has some limitations. First, the small sample size and its uneven distribution limited the broad representativeness of this study. Second, we did not investigate the relationship between molecular characteristics of the *S. aureus* isolates and clinical information (e.g., mortality, severity, complication), which will be the focus in further research. Third, the analysis of genetic relatedness and neighborhood of 555 *S. aureus* isolates was deficient. The PFGE will be carried out in the subsequent experiments. Fourth, our hypothesis of the special ST630 lineage has not been experimentally tested. PCR screening was only capable of detecting the presence of WTA glycosyltransferase genes, but it couldn’t reflect the actual relative abundances of the glycosyltransferases. RNA-seq and NMR spectroscopy will be performed to analyze the WTA phenotypes in the future.

## Conclusion

This study aimed at providing information on epidemiological and molecular characteristics, along with WTA glycosyltransferase profiles of *S. aureus* among different regions. Our results suggest that the prevalence of *S. aureus* and MRSA is dynamic due to differences in geographical regions, and future systematic surveillance continues to be required to better characterize the cause of these differences. The *tarP* gene was present in more clone complexes than just CC5 and CC398. We also found an unusual ST630 lineage, which might present unique WTA structures. Further studies will focus on the functional difference among variable WTA structures and glycosylation patterns.

## Data Availability Statement

All datasets presented in this study are included in the article/Supplementary Material.

## Ethics Statement

All procedures performed were approved by the Institutional Ethics Board of Zhongnan Hospital of Wuhan University (No. 2019126). The present study was a retrospective study focusing on bacteria and did not contain any sensitive personal information. Therefore, informed consent was not required in line with local legislation.

## Author Contributions

YL, TH, and JL contributed to the conception, designed the studies, and obtained funding. MX performed the experiments and wrote the manuscript. MX, XL, and JuZ performed the statistical analysis. YL and YP contributed to manuscript revision. TH, WW, LW, and XX contributed the materials. All authors read and approved the submitted version.

## Conflict of Interest

The authors declare that the research was conducted in the absence of any commercial or financial relationships that could be construed as a potential conflict of interest.
