# Supplementary material for: Molecular Characteristics, Virulence Gene and Wall Teichoic Acid Glycosyltransferase Profiles of Staphylococcus aureus: A Multicenter Study in China
Source: Front Microbiol. 2020 Aug 19;11:2013. doi: 10.3389/fmicb.2020.02013 (PMC7466653; doi:10.3389/fmicb.2020.02013)
Supplement: Supplementary file 1 [file Table_1.DOCX]

**SUPPLEMENTAL MATERIAL**

Table S1. All the primers used for PCR in this study

|  | primers sequence (5' –3') | Production size (bp) | Annealing temperature |
| --- | --- | --- | --- |
| *spa* | F: TAAAGACGATCCTTCGGTGAGC | - | 53℃ |
|  | R: CAGCAGTAGTGCCGTTTGCTT |  |  |
| *arcC* | F: TTGATTCACCAGCGCGTATTGTC | 456 | 55℃ |
|  | R: AGGTATCTGCTTCAATCAGCG |  |  |
| *aroE* | F: ATCGGAAATCCTATTTCACATTC | 456 | 55℃ |
|  | R: GGTGTTGTATTAATAACGATATC |  |  |
| *tpi* | F: TCGTTCATTCTGAACGTCGTGAA | 402 | 55℃ |
|  | R: TTTGCACCTTCTAACAATTGTAC |  |  |
| *glpF* | F: CTAGGAACTGCAATCTTAATCC | 465 | 55℃ |
|  | R: TGGTAAAATCGCATGTCCAATTC |  |  |
| *gmk* | F: ATCGTTTTATCGGGACCATC | 429 | 55℃ |
|  | R: TCATTAACTACAACGTAATCGTA |  |  |
| *pta* | F: GTTAAAATCGTATTACCTGAAGG | 474 | 55℃ |
|  | R: GACCCTTTTGTTGAAAAGCTTAA |  |  |
| *yqiL* | F: CAGCATACAGGACACCTATTGGC | 516 | 55℃ |
|  | R: CGTTGAGGAATCGATACTGGAAC |  |  |
| *pvl* | F: TGCCAGACAATGAATTACCCCCATT | 894 | 57℃ |
|  | R: TCTGCCATATGGTCCCCAACCA |  |  |
| *eta* | F: CGCTGCGGACATTCCTACATGG | 676 | 57℃ |
|  | R: TACATGCCCGCCACTTGCTTGT |  |  |
| *tst* | F: AGCCCTGCTTTTACAAAAGGGGAAAA | 306 | 57℃ |
|  | R: CCAATAACCACCCGTTTTATCGCTTG |  |  |
| *sdrC* | F: CGCATGGCAGTGAATACTGTTGCAGC | 725 | 57℃ |
|  | R: GAAGTATCAGGGGTGAAACTATCCACAAATTG |  |  |
| *sdrD* | F: CCACTGGAAATAAAGTTGAAGTTTCAACTGCC | 467 | 57℃ |
|  | R: CCTGATTTAACTTTGTCATCAACTGTAATTTGTG |  |  |
| *sdrE* | F: GCAGCAGCGCATGACGGTAAAG | 894 | 57℃ |
|  | R: GTCGCCACCGCCAGTGTCATTA |  |  |
| *etb* | F: CAGATAAAGAGCTTTATACACACATTAC | 612 | 57℃ |
|  | R: AGTGAACTTATCTTTCTATTGAAAAACACTC |  |  |
| *hla* | F: GCGAAGAAGGTGCTAACAAAAGT | 379 | 57℃ |
|  | R: AGTTGATTGCCATATACCGGGTT |  |  |
| *fnbB* | F: GTAACAGCTAATGGTCGAATTGATACT | 524 | 57℃ |
|  | R: CAAGTTCGATAGGAGTACTATGTTC |  |  |
| *clfA* | F: AGCGCAAAATCCAGCACAAC | 977 | 55℃ |
|  | R: TACCTGTTAAAACCGGCGCA |  |  |
| *hlg* | F: AAATGGGGCGTGACTCAAAA | 612 | 55℃ |
|  | R: TTCAAATTCGCTCGTGTCGC |  |  |
| *hlb* | F: GTGCACTTACTGACAATAGTGC | 309 | 55℃ |
|  | R: GTTGATGAGTAGCTACCTTCAGT |  |  |
| *clfB* | F: AGTCTTGCAGTTGCTGAACC | 957 | 55℃ |
|  | R: TTTACCTGTCGCTGGGTCAAT |  |  |
| *fnbA* | F: GTGAAAAACAATCTTAGGTACGGCA | 902 | 55℃ |
|  | R: AGTTCAGCCGTTACATCAACCT |  |  |
| *cna* | F: CGATTAAAGTGGCATGGCCG | 947 | 55℃ |
|  | R: GTCTCCACTAGGCAACGCTT |  |  |
| *icaA* | F: TGCAGTTGTCGATGTTGGCT | 167 | 55℃ |
|  | R: ATCTCACGCGTTGCTTCCAA |  |  |
| *arcA* | F: GAGCCAGAAGTACGCGAG | 724 | 55℃ |
|  | R: CACGTAACTTGCTAGAACGAG |  |  |
| *opp3AB* | F: GCAAATCTGTAAATGGTCTGTT | 1183 | 55℃ |
|  | R: GAAGATTGGCAGCACAAAGTG |  |  |
| *tagN* | F: GGGTTAACGGTAAAAGGCCAC | 695 | 57℃ |
|  | R: ATTGATGCCTTCGGCGACTT |  |  |
| *mecA* | F: AAAATCGATGGTAAAGGTTGGC | 533 | 52℃ |
|  | R: AGTTCTGCAGTACCGGATTTGC |  |  |
| *tarS* | F: ATGATGAAATTTTCAGTAATAGTTCCAACATACAA | 1722 | 52℃ |
|  | R: TTATTTTAGCGAGTAAGTCATATGTGCAGT |  |  |
| *tarM* | F: ATGAAAAAAATATTTATGATGGTACATGAGTTAGA | 1482 | 50℃ |
|  | R: TTAGCTATTGAAAAGATTTAACCATTTTTCTAATA |  |  |
| *tarP* | F: ATGAAAAAAGTAAGTGTTATAATGCCAACATTC | 984 | 46℃ |
|  | R: CTATAATAGCTTATCTGCAATCATCACAGC |  |  |

Table S2. Molecular characteristics of *S. aureus* isolates

| Clone complex(no.) | ST(no.) | spa(no.) | MRSA(no.) | | MSSA(no.) | |
| --- | --- | --- | --- | --- | --- | --- |
|  |  |  | invasive | non-invasive | invasive | non-invasive |
| CC1(58) | ST1(18) | t114(4) |  | 4 |  |  |
|  |  | t127(12) |  |  | 4 | 8 |
|  |  | t1784(1) |  |  |  | 1 |
|  |  | t398(1) |  |  |  | 1 |
|  | ST9(1) | t899(1) |  | 1 |  |  |
|  | ST188(38) | t189(33) |  | 1 | 3 | 29 |
|  |  | t2174(1) |  |  |  | 1 |
|  |  | t2765(1) |  |  |  | 1 |
|  |  | t2769(1) |  |  |  | 1 |
|  |  | t416(1) |  |  |  | 1 |
|  |  | t7629(1) |  |  |  | 1 |
|  | ST573(1) | t3876(1) |  |  |  | 1 |
| CC5(130) | ST5(94) | t002(25) |  | 7 | 2 | 16 |
|  |  | t1265(1) |  |  |  | 1 |
|  |  | t1793(1) |  |  |  | 1 |
|  |  | t214(1) |  |  | 1 |  |
|  |  | t2358(1) |  | 1 |  |  |
|  |  | t2460(40) | 8 | 31 |  | 1 |
|  |  | t264(1) |  | 1 |  |  |
|  |  | t2980(1) |  |  |  | 1 |
|  |  | t311(7) | 1 | 6 |  |  |
|  |  | t3794(1) |  |  |  | 1 |
|  |  | t5353(1) |  | 1 |  |  |
|  |  | t548(6) |  | 1 | 1 | 4 |
|  |  | t640(1) |  |  | 1 |  |
|  |  | t777(1) |  |  |  | 1 |
|  |  | t8660(1) |  |  |  | 1 |
|  |  | t9353(2) |  | 2 |  |  |
|  |  | t9476(1) |  |  |  | 1 |
|  |  | t954(2) |  |  |  | 2 |
|  | ST6(24) | t1131(1) |  |  |  | 1 |
|  |  | t14287(1) |  |  |  | 1 |
|  |  | t3802(1) |  |  |  | 1 |
|  |  | t4676(1) |  |  |  | 1 |
|  |  | t701(20) |  |  | 3 | 17 |
|  | ST672(1) | t014(1) |  |  |  | 1 |
|  | ST764(5) | t002(2) |  | 2 |  |  |
|  |  | t1084(2) |  | 2 |  |  |
|  |  | t601(1) |  | 1 |  |  |
|  | ST950(1) | t895(1) |  |  |  | 1 |
|  | ST965(3) | t653(1) |  |  |  | 1 |
|  |  | t062(2) |  | 1 |  | 1 |
|  | ST3687(2) | t5166(2) |  |  |  | 2 |
| CC7(33) | ST7(33) | t091(22) | 1 | 3 | 2 | 16 |
|  |  | t3884(1) |  |  |  | 1 |
|  |  | t605(1) |  |  |  | 1 |
|  |  | t6835(1) |  |  |  | 1 |
|  |  | t796(8) |  | 1 |  | 7 |
| CC8(63) | ST8(3) | t024(1) |  | 1 |  |  |
|  |  | t7087(2) |  |  |  | 2 |
|  | ST72(7) | t148(5) |  |  | 1 | 4 |
|  |  | t3092(1) |  |  |  | 1 |
|  |  | t664(1) |  | 1 |  |  |
|  | ST239(41) | t030(33) | 4 | 27 |  | 2 |
|  |  | t037(2) |  | 1 |  | 1 |
|  |  | t4516(1) |  | 1 |  |  |
|  |  | t1510(1) |  | 1 |  |  |
|  |  | t459(4) |  | 4 |  |  |
|  | ST630(12) | t2196(1) |  | 1 |  |  |
|  |  | t377(5) |  |  | 1 | 4 |
|  |  | t4047(1) |  |  |  | 1 |
|  |  | t4549(5) | 1 | 4 |  |  |
| CC15(14) | ST15(9) | t084(5) |  | 1 | 1 | 3 |
|  |  | t120(1) |  |  |  | 1 |
|  |  | t12513(1) |  |  |  | 1 |
|  |  | t14014(1) |  |  |  | 1 |
|  |  | t774(1) |  |  |  | 1 |
|  | ST845(5) | t084(2) |  |  |  | 2 |
|  |  | t346(3) |  |  |  | 3 |
| CC20(6) | ST1281(6) | t164(6) |  |  | 1 | 5 |
| CC22(54) | ST22(47) | t16061(1) |  | 1 |  |  |
|  |  | t309(42) |  | 4 | 1 | 37 |
|  |  | t5763(3) |  |  |  | 3 |
|  |  | t8202(1) |  | 1 |  |  |
|  | ST217(6) | t13828(2) |  | 1 |  | 1 |
|  |  | t309(4) |  | 1 |  | 3 |
|  | ST3306(1) | t309(1) |  |  |  | 1 |
| CC25(14) | ST25(14) | t078(8) |  | 1 |  | 7 |
|  |  | t081(1) |  |  |  | 1 |
|  |  | t280(1) |  |  |  | 1 |
|  |  | t3033(1) |  |  |  | 1 |
|  |  | t528(1) |  |  |  | 1 |
|  |  | t7642(1) |  |  |  | 1 |
|  |  | t9188(1) |  |  |  | 1 |
| CC30(8) | ST30(5) | NT(1) |  |  |  | 1 |
|  |  | t338(4) |  |  |  | 4 |
|  | ST1163(2) | t021(2) |  |  |  | 2 |
|  | ST2580(1) | t3351(1) |  | 1 |  |  |
| CC45(12) | ST45(10) | t026(1) | 1 |  |  |  |
|  |  | t116(7) | 2 | 5 |  |  |
|  |  | t1510(1) |  | 1 |  |  |
|  |  | t157(1) |  | 1 |  |  |
|  | ST3154(1) | t630(1) |  |  |  | 1 |
|  | ST508(1) | t9058(1) |  | 1 |  |  |
| CC59(94) | ST59(88) | t163(7) |  | 4 |  | 3 |
|  |  | t172(16) |  | 14 | 1 | 1 |
|  |  | t1751(1) |  | 1 |  |  |
|  |  | t2365(1) |  | 1 |  |  |
|  |  | t3523(1) |  | 1 |  |  |
|  |  | t3527(2) |  | 1 |  | 1 |
|  |  | t3592(2) |  | 2 |  |  |
|  |  | t437(54) | 5 | 39 | 2 | 8 |
|  |  | t441(3) |  | 2 |  | 1 |
|  |  | t5795(1) |  | 1 |  |  |
|  | ST338(6) | t13774(3) |  | 3 |  |  |
|  |  | t3590(1) |  | 1 |  |  |
|  |  | t437(2) |  | 1 |  | 1 |
| CC88(14) | ST88(14) | t1376(7) |  |  | 2 | 5 |
|  |  | t18556(1) |  |  |  | 1 |
|  |  | t2592(1) |  | 1 |  |  |
|  |  | t3155(1) |  |  |  | 1 |
|  |  | t3622(3) | 1 | 1 |  | 1 |
|  |  | t4333(1) |  |  |  | 1 |
| CC97(2) | ST97(1) | t267(1) |  |  | 1 |  |
|  | ST464(1) | t3904(1) |  |  |  | 1 |
| CC121(9) | ST121(9) | t021(1) |  |  |  | 1 |
|  |  | t1425(1) |  |  |  | 1 |
|  |  | t14752(1) |  |  |  | 1 |
|  |  | t2019(2) |  |  |  | 2 |
|  |  | t2086(1) |  |  | 1 |  |
|  |  | t2091(2) |  |  |  | 2 |
|  |  | t954(1) |  |  |  | 1 |
| CC291(1) | ST291(1) | t2313(1) |  | 1 |  |  |
| CC398(41) | ST398(41) | t011(5) |  | 1 |  | 4 |
|  |  | t034(17) |  | 3 | 1 | 13 |
|  |  | t1451(4) |  | 1 |  | 3 |
|  |  | t1580(1) |  |  |  | 1 |
|  |  | t18621(1) |  |  |  | 1 |
|  |  | t2370(1) |  | 1 |  |  |
|  |  | t2765(1) |  |  |  | 1 |
|  |  | t3625(1) |  |  |  | 1 |
|  |  | t5462(1) |  | 1 |  |  |
|  |  | t5635(1) |  | 1 |  |  |
|  |  | t571(8) |  |  |  | 8 |
| CC509(2) | ST509(2) | t375(2) |  | 1 |  | 1 |

Table S3. Molecular characteristics of *S. aureus* isolates among different regions

| CC(no.) | northern China(126) | | | | southern China(144) | | | | central China(285) | | | |
| --- | --- | --- | --- | --- | --- | --- | --- | --- | --- | --- | --- | --- |
|  | ST(no.) | spa(no.) | MRSA(no.) | MSSA(no.) | ST(no.) | spa(no.) | MRSA(no.) | MSSA(no.) | ST(no.) | spa(no.) | MRSA(no.) | MSSA(no.) |
| CC1(58) | ST1(2) | t114(1) | 1 |  | ST1(7) | t114(2) | 2 |  | ST1(9) | t1784(1) |  | 1 |
|  |  | t398(1) |  | 1 |  | t127(5) |  | 5 |  | t127(7) |  | 7 |
|  | ST188(2) | t189(1) | 1 |  | ST188(9) | t189(8) |  | 8 |  | t114(1) | 1 |  |
|  |  | t7629(1) |  | 1 |  | t2174(1) |  | 1 | ST188(27) | t2765(1) |  | 1 |
|  |  |  |  |  | ST9(1) | t899(1) | 1 |  |  | t189(24) |  | 24 |
|  |  |  |  |  |  |  |  |  |  | t2769(1) |  | 1 |
|  |  |  |  |  |  |  |  |  |  | t416(1) |  | 1 |
|  |  |  |  |  |  |  |  |  | ST573(1) | t3876(1) |  | 1 |
| CC5(130) | ST5(19) | t002(7) |  | 7 | ST5(15) | t002(10) | 6 | 4 | ST5(60) | t002(8) | 1 | 7 |
|  |  | t2460(8) | 7 | 1 |  | t1265(1) |  | 1 |  | t1793(1) |  | 1 |
|  |  | t264(1) | 1 |  |  | t214(1) |  | 1 |  | t2460(32) | 32 |  |
|  |  | t548(1) | 1 |  |  | t2358(1) | 1 |  |  | t311(7) | 7 |  |
|  |  | t9353(2) | 2 |  |  | t2980(1) |  | 1 |  | t3794(1) |  | 1 |
|  | ST6(12) | t14287(1) |  | 1 |  | t777(1) |  | 1 |  | t5353(1) | 1 |  |
|  |  | t3802(1) |  | 1 | ST6(3) | t701(3) |  | 3 |  | t548(5) |  | 5 |
|  |  | t701(9) |  | 9 | ST764(1) | t1084(1) | 1 |  |  | t640(1) |  | 1 |
|  |  | t4676(1) |  | 1 |  |  |  |  |  | t8660(1) |  | 1 |
|  | ST965(2) | t062(1) |  | 1 |  |  |  |  |  | t9476(1) |  | 1 |
|  |  | t653(1) |  | 1 |  |  |  |  |  | t954(2) |  | 2 |
|  | ST3687(2) | t5166(2) |  | 2 |  |  |  |  | ST6(9) | t701(8) |  | 8 |
|  |  |  |  |  |  |  |  |  |  | t1131(1) |  | 1 |
|  |  |  |  |  |  |  |  |  | ST672(1) | t014(1) |  | 1 |
|  |  |  |  |  |  |  |  |  | ST764(4) | t1084(1) | 1 |  |
|  |  |  |  |  |  |  |  |  |  | t002(2) | 2 |  |
|  |  |  |  |  |  |  |  |  |  | t601(1) | 1 |  |
|  |  |  |  |  |  |  |  |  | ST950(1) | t895(1) |  | 1 |
|  |  |  |  |  |  |  |  |  | ST965(1) | t062(1) | 1 |  |
| CC7(33) | ST7(3) | t091(2) | 1 | 1 | ST7(6) | t091(6) | 2 | 4 | ST7(24) | t091(14) | 1 | 13 |
|  |  | t796(1) |  | 1 |  |  |  |  |  | t3884(1) |  | 1 |
|  |  |  |  |  |  |  |  |  |  | t605(1) |  | 1 |
|  |  |  |  |  |  |  |  |  |  | t6835(1) |  | 1 |
|  |  |  |  |  |  |  |  |  |  | t796(7) | 1 | 6 |
| CC8(63) | ST239(5) | t030(4) | 3 | 1 | ST239(4) | t030(2) | 2 |  | ST8(3) | t024(1) | 1 |  |
|  |  | t4516(1) | 1 |  |  | t037(2) | 1 | 1 |  | t7087(2) |  | 2 |
|  | ST630(3) | t377(2) |  | 2 | ST630(1) | t377(1) |  | 1 | ST72(5) | t664(1) | 1 |  |
|  |  | t4549(1) | 1 |  |  |  |  |  |  | t148(3) |  | 3 |
|  | ST72(2) | t148(2) |  | 2 |  |  |  |  |  | t3092(1) |  | 1 |
|  |  |  |  |  |  |  |  |  | ST239(32) | t030(27) | 26 | 1 |
|  |  |  |  |  |  |  |  |  |  | t1510(1) | 1 |  |
|  |  |  |  |  |  |  |  |  |  | t459(4) | 4 |  |
|  |  |  |  |  |  |  |  |  | ST630(8) | t2196(1) | 1 |  |
|  |  |  |  |  |  |  |  |  |  | t377(2) |  | 2 |
|  |  |  |  |  |  |  |  |  |  | t4549(4) | 4 |  |
|  |  |  |  |  |  |  |  |  |  | t4047(1) |  | 1 |
| CC15(14) | ST15(1) | t774(1) |  | 1 |  |  |  |  | ST15(8) | t084(5) | 1 | 4 |
|  | ST845(4) | t084(2) |  | 2 |  |  |  |  |  | t14014(1) |  | 1 |
|  |  | t346(2) |  | 2 |  |  |  |  |  | t12513(1) |  | 1 |
|  |  |  |  |  |  |  |  |  |  | t120(1) |  | 1 |
|  |  |  |  |  |  |  |  |  | ST845(1) | t346(1) |  | 1 |
| CC20(6) |  |  |  |  | ST1281(1) | t164(1) |  | 1 | ST1281(5) | t164(5) |  | 5 |
| CC22(54) | ST22(24) | t309(22) |  | 22 | ST22(17) | t309(14) | 3 | 11 | ST22(6) | t309(6) | 1 | 5 |
|  |  | t5763(2) |  | 2 |  | t16061(1) | 1 |  | ST217(6) | t309(4) | 1 | 3 |
|  |  |  |  |  |  | t8202(1) | 1 |  |  | t13828(2) | 1 | 1 |
|  |  |  |  |  |  | t5763(1) |  | 1 |  |  |  |  |
|  |  |  |  |  | ST3306(1) | t309(1) |  | 1 |  |  |  |  |
| CC25(14) | ST25(6) | t078(6) | 1 | 5 | ST25(1) | t9188(1) |  | 1 | ST25(7) | t078(2) |  | 2 |
|  |  |  |  |  |  |  |  |  |  | t528(1) |  | 1 |
|  |  |  |  |  |  |  |  |  |  | t280(1) |  | 1 |
|  |  |  |  |  |  |  |  |  |  | t7642(1) |  | 1 |
|  |  |  |  |  |  |  |  |  |  | t3033(1) |  | 1 |
|  |  |  |  |  |  |  |  |  |  | t081(1) |  | 1 |
| CC30(8) | ST30(2) | t338(2) |  | 2 | ST2580(1) | t3351(1) | 1 |  | ST1163(2) | t021(2) |  | 2 |
|  |  |  |  |  | ST30(3) | NT(1) |  | 1 |  |  |  |  |
|  |  |  |  |  |  | t338(2) |  | 2 |  |  |  |  |
| CC45(12) | ST3154(1) | t630(1) |  | 1 | ST45(6) | t116(4) | 4 |  | ST45(4) | t1510(1) | 1 |  |
|  |  |  |  |  |  | t026(1) | 1 |  |  | t116(3) | 3 |  |
|  |  |  |  |  |  | t157(1) | 1 |  | ST508(1) | t9058(1) | 1 |  |
| CC59(94) | ST59(24) | t163(2) |  | 2 | ST59(34) | t163(5) | 4 | 1 | ST59(30) | t172(5) | 5 |  |
|  |  | t172(1) | 1 |  |  | t172(10) | 8 | 2 |  | t1751(1) | 1 |  |
|  |  | t437(21) | 19 | 2 |  | t2365(1) | 1 |  |  | t3523(1) | 1 |  |
|  |  |  |  |  |  | t3592(2) | 2 |  |  | t3527(2) | 1 | 1 |
|  |  |  |  |  |  | t437(14) | 11 | 3 |  | t437(19) | 14 | 5 |
|  |  |  |  |  |  | t441(1) | 1 |  |  | t441(2) | 1 | 1 |
|  |  |  |  |  |  | t5795(1) | 1 |  | ST338(1) | t437(1) | 1 |  |
|  |  |  |  |  | ST338(5) | t13774(3) | 3 |  |  |  |  |  |
|  |  |  |  |  |  | t3590(1) | 1 |  |  |  |  |  |
|  |  |  |  |  |  | t437(1) |  | 1 |  |  |  |  |
| CC88(14) | ST88(3) | t1376(3) |  | 3 | ST88(6) | t1376(3) |  | 3 | ST88(5) | t18556(1) |  | 1 |
|  |  |  |  |  |  | t2592(1) | 1 |  |  | t1376(1) |  | 1 |
|  |  |  |  |  |  | t3622(1) |  | 1 |  | t3622(2) | 2 |  |
|  |  |  |  |  |  | t4333(1) |  | 1 |  | t3155(1) |  | 1 |
| CC97(2) |  |  |  |  | ST97(1) | t267(1) |  | 1 |  |  |  |  |
|  |  |  |  |  | ST464(1) | t3904(1) |  | 1 |  |  |  |  |
| CC121(9) | ST121(1) | t14752(1) |  | 1 |  |  |  |  | ST121(8) | t2019(2) |  | 2 |
|  |  |  |  |  |  |  |  |  |  | t2086(1) |  | 1 |
|  |  |  |  |  |  |  |  |  |  | t2091(2) |  | 2 |
|  |  |  |  |  |  |  |  |  |  | t1425(1) |  | 1 |
|  |  |  |  |  |  |  |  |  |  | t954(1) |  | 1 |
|  |  |  |  |  |  |  |  |  |  | t021(1) |  | 1 |
| CC291(1) | ST291(1) | t2313(1) | 1 |  |  |  |  |  |  |  |  |  |
| CC398(41) | ST398(7) | t034(1) | 1 |  | ST398(18) | t011(4) |  | 4 | ST398(16) | t034(7) | 1 | 6 |
|  |  | t1451(3) | 1 | 2 |  | t034(9) | 1 | 8 |  | t5462(1) | 1 |  |
|  |  | t571(2) |  | 2 |  | t18621(1) |  | 1 |  | t1580(1) |  | 1 |
|  |  | t011(1) | 1 |  |  | t2370(1) | 1 |  |  | t2765(1) |  | 1 |
|  |  |  |  |  |  | t571(3) |  | 3 |  | t571(3) |  | 3 |
|  |  |  |  |  |  |  |  |  |  | t5635(1) | 1 |  |
|  |  |  |  |  |  |  |  |  |  | t3625(1) |  | 1 |
|  |  |  |  |  |  |  |  |  |  | t1451(1) |  | 1 |
| CC509(2) |  |  |  |  | ST509(2) | t375(2) | 1 | 1 |  |  |  |  |

Table S4. WTA glycosylation patterns

| WTA glycosylation patterns(no.) | ST(no.) | MRSA(no.) | MSSA(no.) |
| --- | --- | --- | --- |
| *tarP*+/*tarS*+/*tarM*+/*tagN*-(2) | ST845(2) |  | 2 |
| *tarP*+/*tarS*+/*tarM*-/*tagN*-(14) | ST5(2) |  | 2 |
|  | ST7(5) | 2 | 3 |
|  | ST188(4) |  | 4 |
|  | ST398(2) |  | 2 |
|  | ST464(1) |  | 1 |
| *tarP*-/*tarS*+/*tarM*+/*tagN*-(98) | ST1(17) | 4 | 13 |
|  | ST5(1) |  | 1 |
|  | ST6(1) |  | 1 |
|  | ST7(1) |  | 1 |
|  | ST8(3) | 1 | 2 |
|  | ST15(9) | 1 | 8 |
|  | ST22(2) |  | 2 |
|  | ST30(5) |  | 5 |
|  | ST59(1) | 1 |  |
|  | ST121(8) |  | 8 |
|  | ST188(1) |  | 1 |
|  | ST217(1) |  | 1 |
|  | ST238(39) | 37 | 2 |
|  | ST573(1) |  | 1 |
|  | ST630(2) |  | 2 |
|  | ST845(3) |  | 3 |
|  | ST1163(2) |  | 2 |
|  | ST2580(1) | 1 |  |
| *tarP*-/*tarS*+/*tarM*-/*tagN*-(431) | ST1(1) |  | 1 |
|  | ST5(91) | 59 | 32 |
|  | ST6(23) |  | 23 |
|  | ST7(27) | 3 | 24 |
|  | ST9(1) | 1 |  |
|  | ST22(45) | 6 | 39 |
|  | ST25(14) | 1 | 13 |
|  | ST45(10) | 10 |  |
|  | ST59(87) | 70 | 17 |
|  | ST72(7) | 1 | 6 |
|  | ST88(14) | 3 | 11 |
|  | ST97(1) |  | 1 |
|  | ST121(1) |  | 1 |
|  | ST188(33) | 1 | 32 |
|  | ST217(5) | 2 | 3 |
|  | ST239(2) | 1 | 1 |
|  | ST291(1) | 1 |  |
|  | ST338(6) | 5 | 1 |
|  | ST398(39) | 8 | 31 |
|  | ST508(1) | 1 |  |
|  | ST509(2) | 1 | 1 |
|  | ST672(1) |  | 1 |
|  | ST764(5) | 5 |  |
|  | ST950(1) |  | 1 |
|  | ST965(3) | 1 | 2 |
|  | ST1281(6) |  | 6 |
|  | ST3154(1) |  | 1 |
|  | ST3306(1) |  | 1 |
|  | ST3687(2) |  | 2 |
| *tarP*-/*tarS*-/*tarM*+/*tagN*+(10) | ST630(10) | 6 | 4 |
